# Comparable outcomes of allogeneic peripheral blood versus bone marrow hematopoietic stem cell transplantation from a sibling donor for pediatric patients

**DOI:** 10.1007/s00277-024-05737-5

**Published:** 2024-04-10

**Authors:** Bo Kyung Kim, Kyung Taek Hong, Jung Yoon Choi, Hyery Kim, Hyun Jin Park, Hyoung Jin Kang

**Affiliations:** 1 Department of Pediatrics, Seoul National University College of Medicine, Seoul National University Cancer Research Institute, 101 Daehak-ro, Jongno-gu, Seoul, 03722 Korea; 2 Seoul National University Cancer Research Institute, Seoul, Korea; 3 Divison of Pediatric Hematology/Oncology, Department of Pediatrics, Children’s Hospital, Asan Medical Center, University of Ulsan College of Medicine, Seoul, Korea

**Keywords:** Sibling ematopoietic stem cell transplantation, Bone marrow, Peripheral blood stem cells

## Abstract

Traditionally, bone marrow (BM) has been preferred as a source of stem cells (SCs) in pediatric hematopoietic SC transplantation (HSCT); however, the use of peripheral blood SCs (PBSC) has recently increased. With advancing graft-versus-host disease (GVHD) prophylaxis, whether the BM is still a better SC source than PB in sibling donor HSCT remains controversial. Here, we compared the results of BM transplantation (BMT) and PBSC transplantation (PBSCT) in pediatric patients with malignant or non-malignant diseases receiving sibling HSCT using a total of 7.5 mg/kg of anti-thymocyte globulin (ATG). We retrospectively reviewed children who received HSCT from a sibling donor between 2005 and 2020 at Seoul National University Children’s Hospital. Of the 86 patients, 40 underwent BMT, and 46 underwent PBSCT. Fifty- six patients had malignant diseases, whereas thirty patients had non-malignant diseases. All conditioning regimens comprised ATG. Busulfan-based myeloablative conditioning regimens were administered to patients with malignant diseases and approximately half of those with non-malignant diseases. The remaining half of the patients with non-malignant diseases were administered cyclophosphamide-based reduced- intensity conditioning regimens. According to studies conducted at our center, all BM donors received G-CSF before harvest to achieve early engraftment. In all 86 patients (47 males and 39 females), the median age at the time of HSCT was 11.4 (range, 0.7 − 24.6) years. The median follow-up period was 57.9 (range, 0.9–228.6) months, and the corresponding values for those with BM and PBSC were 77 (range, 2.4–228.6) months and 48.7 (range, 0.9–213.2) months, respectively. Engraftment failure occurred in one patient with BM and no patient with PBSC. The cumulative incidence of acute GVHD with grades II–IV was higher in PBSC (BM 2.5%, PBSC 26.1%, *p* = 0.002), but there was no significant difference in those with grades III–IV acute GVHD (BM 0%, PBSC 6.5%, *p* = 0.3703) and extensive chronic GVHD (BM 2.5%, PBSC 11.6%, *p* = 0.1004). There were no significant differences in treatment-related mortality (TRM) (BM 14.2%, PBSC 6.8%, *p* = 0.453), 5-year event-free survival (EFS) (BM 71.5%, PBSC 76.2%, *p* = 0.874), and overall survival (OS) rates (BM 80.8%, PBSC 80.3%, *p* = 0.867) between BM and PBSC in the univariate analysis. In the multivariate analysis, which included all factors with *p* < 0.50 in the univariate analysis, there was no significant prognostic factor for EFS or OS. There was no significant difference in the relapse incidence between BM and PBSC among patients with malignant diseases (BM 14.2%, PBSC 6.8%, *p* = 0.453). Additionally, there were no significant differences in the TRM, 5-year EFS, and OS rates between malignant and non-malignant diseases nor between the busulfan-based myeloablative regimen and reduced-intensity chemotherapy using cyclophosphamide. In this study, we showed no significant differences in EFS, OS, TRM, and GVHD, except for acute GVHD grades II–IV, between BMT and PBSCT from sibling donors, using ATG (a total of 7.5 mg/kg). Therefore, PB collection, which is less invasive for donors and less labor-intensive for doctors, could also be considered an acceptable SC source for sibling donor HSCT in children.

## Introduction

Hematopoietic stem cell transplantation (HSCT) is an essential treatment for patients with hematologic malignancies or immunodeficiency. The results of allogeneic HSCT have improved, the number of survivors has increased, and the sources of transplants have recently changed. Traditionally, the bone marrow (BM) has been a common SC source in pediatric HSCT. However, peripheral blood SCs (PBSC), mobilized by granulocyte colony-stimulating factor, have been used as an alternative SC source from the early 2000s [1]. Additionally, the use of PBSC transplantation (PBSCT) has gradually increased owing to the following disadvantages of BM transplantation (BMT): difficulty in finding donors, complicated procedures, and donor complications. To date, the results of PBSCT have not been comparable to those of BMT [2–4]. However, with advances in graft-versus-host disease (GVHD) prophylaxis, whether BM is still a better SC source than PB in sibling donor HSCT remains controversial.

A recent systematic review and meta-analysis comparing BMT and PBSCT for hematologic malignancies in children concluded that overall survival (OS) and event-free survival (EFS) were comparable between both transplantation types (PBSCT, 56.2%; BMT, 63.5%; relative risk [RR], 1.17; 95% confidence interval, 0.91 to 1.52 for OS; PBSCT, 49.9%; BMT, 57.2%; RR, 1.14; 95% confidence interval, 0.93 to 1.39 for EFS), but non-relapse mortality and chronic GVHD were slightly higher in the PBSC group than in the BMT group (RR, 1.73; 95% confidence interval, 1.50 to 1.99 versus RR, 1.55; 95% confidence interval, 1.18 to 2.03) [5].

No studies have compared BM and PBSC in children, including those with malignant and non-malignant diseases. Here, we compared the results of BMT and PBSCT in pediatric patients with malignant or non-malignant disease receiving sibling HSCT using a total of 7.5 mg/kg of anti-thymocyte globulin (ATG).

## Methods

### Study population and study design

We retrospectively reviewed children who received HSCT from a sibling donor between 2005 and 2020 at Seoul National University Children’s Hospital. Patients below 25 years of age who underwent SCT were included.

### Donor selection

For donor selection, HLA-A, -B, -C, and –DRB1 matching was confirmed using a high-resolution molecular method for all patients and donors. All the patients received the designated conditioning regimen after providing informed consent.

### Transplantation protocol

Table 1.presents the conditioning regimen for each group. All conditioning regimens comprised ATG (2.5 mg/kg/day, once daily from days − 4 to -2). Busulfan-based myeloablative conditioning (MAC) regimens were administered to patients with malignant diseases and to approximately half of those with non-malignant diseases. The other half of the patients with non-malignant diseases were administered cyclophosphamide-based reduced-intensity conditioning regimens (RICs). According to the studies conducted at our center, all BM donors received G-CSF (10 µg/kg/day for 2 days) prior to harvest to achieve early engraftment [6, 7]

**Table 1:** Details of conditioning regimen

Disease and stem cell source	Conditioning regimen	Number of patients (n)	Details of conditioning regimen
Non-malignant BMT			
	BuFluATG (before 2019)	1	Bu 0.8 mg/kg qid i.v. d-6~d-3; Flu 40 mg/m^2 once daily i.v. d-8~d-3; rATG 2.5 mg/kg i.v. d-4~d-2
	BuFluATG (after 2019)	10	Bu with TDM: Bu (120 mg/m^2 for patients≥1year of age and 80 mg/m^2 for patients<1 year of age) was administered as a starter dose on d-8 and administered once daily thereafter. A subsequent targeted dose of busulfan was analyzed according to the TDM results from d-7 to d-5
	CyFluATG	5	CPM 60 mg/kg i.v. d-8, d-7; Flu 40 mg/m^2 i.v. d-6~d-2; rATG 2.5 mg/kg i.v. d-4~d-2
	CyATGPro	3	Procarbazine 12.5 mg/kg p.o. d-7, d-5, d-3; CPM 50 mg/kg i.v. d-5~d-2; rATG 2.5 mg/kg i.v. d-6, d-4, d-2
	CyATG	2	CPM 50 mg/kg i.v. d-7~ d-4; rATG 2.5 mg/kg i.v. d-6~d-4
	BuFluATG from genetic rare disease	1	Bu with TDM; Flu 40 mg/m^2 i.v. d-8~d-4, rATG 2.5 mg/kg i.v. d-4~ d-2
	BuCyATG	1	Bu with TDM; CPM 60 mg/kg i.v. d-3,d-2; rATG 2.5 mg/kg i.v. d-4~d-2
**Non-malignant PBSCT**			
	BuFluATG	1	Bu with TDM; Flu 40 mg/m^2 i.v. d-8~d-3; rATG 2.5 mg/kg i.v. d-4~d-2)
	BuFluATG from genetic rare disease	1	Bu with TDM; Flu 40 mg/m^2 i.v. d-8~d-4; rATG 2.5 mg/kg i.v. d-4~d-2
	CyFluATG	1	CPM 60 mg/kg i.v. d-8,d-7; Flu 4 0 mg/m^2 i.v. d-7~ d-2; rATG 2.5 mg/kg i.v. d-4~d-2
**Malignant BMT**			
	LiBuCyATG	3	Bu 0.8 mg/kg qid i.v. d-6~d-3; CPM 60 mg/kg once daily i.v. d-3, d-2; rATG 2.5 mg/kg i.v. d-4~ d-2
	BuFluVPATG	2	Bu with TDM, Flu 40 mg/m^2 i.v. d-8~d-3; VP 20 mg/kg i.v. d-4~d-2; rATG 2.5 mg/kg i.v. d-4~d-2
	BuFluATG	1	Bu with TDM, Flu 40 mg/m^2 i.v. d-8~d-3; rATG 2.5 mg/kg i.v. d-4~d-2
	TBIAcCy	1	TBI 300 cGy d-9~d-6; cytarabine 3 g/m^2 bid d-5, d-4; CPM 60 mg/kg iv. d-3, d-2; rATG 2.5 mg/kg i.v. d-4~ d-2
**Malignant PBSCT**			
	BuFluVPATG	29	Bu with TDM, Flu 40 mg/m^2 i.v. d-8~d-3; VP 20 mg/kg i.v. d-4~d-2; rATG 2.5 mg/kg i.v. d-4~d-2
	BuFluATG	13	Bu with TDM, Flu 40 mg/m^2 i.v. d-8~d-3; rATG 2.5 mg/kg i.v. d-4~d-2
	BuCyMelATG	1	Bu with TDM, CPM 60 mg/kg i.v. d-4, d-3; Melphalan 140 mg/m^2 i.v. d-2; rATG 2.5 mg/kg i.v. d-8~ d-6

Bu, busulfan; Flu, fludarabine; rATG, rabbit anti-thymocyte globulin; CPM, cyclophosphamide; VP, etoposide; TDM, therapeutic drug monitoring; i.v., intravenous; p.o. per oral; d, day

### GVHD prophylaxis and supportive care

Most patients were treated with GVHD prophylaxis for related-HSCT with cyclosporine (from day − 2) and prednisolone (from days 7 to 36). Exceptionally, patients who underwent PBSCT for malignant diseases used cyclosporine only after August 2015 for the graft-versus-leukemia (GVL) effect. Cyclosporine was administered until 8 months after HSCT for malignant and non-malignant diseases. Prophylactic treatments for veno-occlusive diseases (VODs) and infections were administered according to our institutional guidelines for HSCT [8].

### Engraftment and toxicities

Neutrophil engraftment was defined as the first of 3 consecutive days on which the absolute neutrophil count was > 0.5 × 10^9^/L, whereas platelet recovery was defined as the day on which the platelet count was > 20 × 10^9^/L without platelet transfusions in the prior 7 days. Bone marrow examination was performed at 1, 3, 6, and 12 months after HSCT, and hematopoietic chimerism was evaluated through molecular analysis of short tandem repeat regions. Regimen-related toxicity, except for GVHD, was graded according to the National Cancer Institute Common Toxicity Criteria (v4.0).

### Statistical analyses

Cumulative incidences (CIs) of acute and chronic GVHD were evaluated using graft failure, relapse, and treatment-related mortality (TRM) as competing risks. The incidence of relapse was evaluated using a CI curve. Events were defined as relapse, TRM, or graft failure. OS and EFS were analyzed using the Kaplan–Meier method. Differences in survival rates were investigated using the log-rank test. Statistical analyses were conducted using R version 3.2.2 and SPSS 25.0.

### Ethics statement

This retrospective study was approved by the Institutional Review Board of Seoul National University Hospital (H-2212-148-1390).

## Results

### Characteristics of the patients

The clinical characteristics of the patients are summarized in Table 2. Of the 86 patients, 40 underwent BMT and 46 underwent PBSCT. Fifty-six patients had malignant diseases, whereas thirty patients had non-malignant diseases. The median age at the time of HSCT was 11.4 (range, 0.7–24.6) years; 47 were male, and 39 were female. Since most malignant patients received PBSCT, most patients with PBSC received MAC (45 of 46 patients), and only one-third of the patients with BM received RICs. The median follow-up period was 57.9 (range, 0.9–228.6) months, and the corresponding values for those with BM and PBSC were 77.0 (range, 2.4–228.6) months and 48.7 (range, 0.9–213.2) months, respectively.

**Table 2:** Patient characteristics (N = 86)

	BM	PB	p-value
	*n*=40	*n*=46	
Age, yr	11.4 (2.2-19.2)	11.1 (0.7-24.6)	0.979
Sex			0.952
Male	22 (55.0)	25 (54.3)	
Female	18 (45.0)	21 (45.7)	
Body weight, kg	38 (11.0-87.3)	40.8 (7.4-76.2)	0.61
Diagnosis			
Acute lymphoblastic leukemia	5 (12.5)	18 (39.1)	
Acute myeloid leukemia	6 (15.0)	19 (41.3)	
Severe aplastic anemia	13 (32.5)	0	
Chronic granulomatous disease	7 (17.5)	0	
Other malignancies*	2 (5.0)	6 (13.0)	
Other nonmalignant diseases@	6 (15.0)	3 (6.5)	
Conditioning regimen			<0.0001
MAC	26 (65.0)	45 (97.8)	
RIC	14 (35.0)	1 (2.2)	
Neutrophil engraftment day	10.0 (8-25)	9 (8-14)	0.009
Platelet engraftment day	19.0 (7-90)	10 (5-54)	0.045
Engraftment failure	1 (2.5)	0 (0)	0.286
Donor-to-recipient sex direction			0.412
Male→Male	13 (32.5)	13 (28.3)	
Male→Female	13 (32.5)	11 (23.9)	
Female→Female	5 (12.5)	10 (21.7)	
Female→Male	9 (22.5)	12 (26.1)	
Donor age	10.0 (2-24)	10.0 (1.5-26)	0.08
Infused TNC, x10^8/kg	8.0 (1.8-23.0)	13.2 (8.0-26.8)	0.799
CD34+ cells, x10^6/kg	4.1 (0.3-31.9)	7.4 (3.1-19.5)	0.036

Data are presented as median (range) or n (%)

BM, bone marrow; PB, peripheral blood; BSA, body surface area; MAC, myeloablative conditioning; RIC, reduced intensity conditioning; TNC, total nuclear cells; MNC, mononuclear cells

### Engraftment

The median numbers of neutrophil and platelet engraftment days were 10.0 (range, 8–25) and 19 (range 7–90) in BM and 9 (range 8–14) and 10 (range 5–54) in PBSC, respectively. Primary engraftment failure occurred in one non-malignant patient with BM whose platelet count did not recover. This patient received a second related HSCT with PB from the patient’s brother 116 days after the first related BMT. Neutrophil and platelet engraftment was achieved on days 11 and 15, respectively, and the patient remained disease-free at the last follow-up, 5 years after the second related PBSCT.

### Complications

Severe VOD was not present in any of the patients. Elevations of aspartate, alanine aminotransferase, and total bilirubin levels of at least grade 3 severity were observed in five patients with BMT (12.5%) and seven patients with PBSCT (15.2%). None of the patients was diagnosed with greater than grade 3 hemorrhagic cystitis. One patient with malignant PBSCT had cytomegalovirus (CMV) disease, which caused CMV enteritis, and the treatment was maintained for approximately 4 weeks. Neutropenic fever occurred in most patients (56.5% of patients with non-malignant BMT, 66.7% of those with non-malignant PBSCT, 85.7% of those with malignant BMT, and 93.0% of those with malignant PBSCT).

### GVHD

**Fig. 1:**
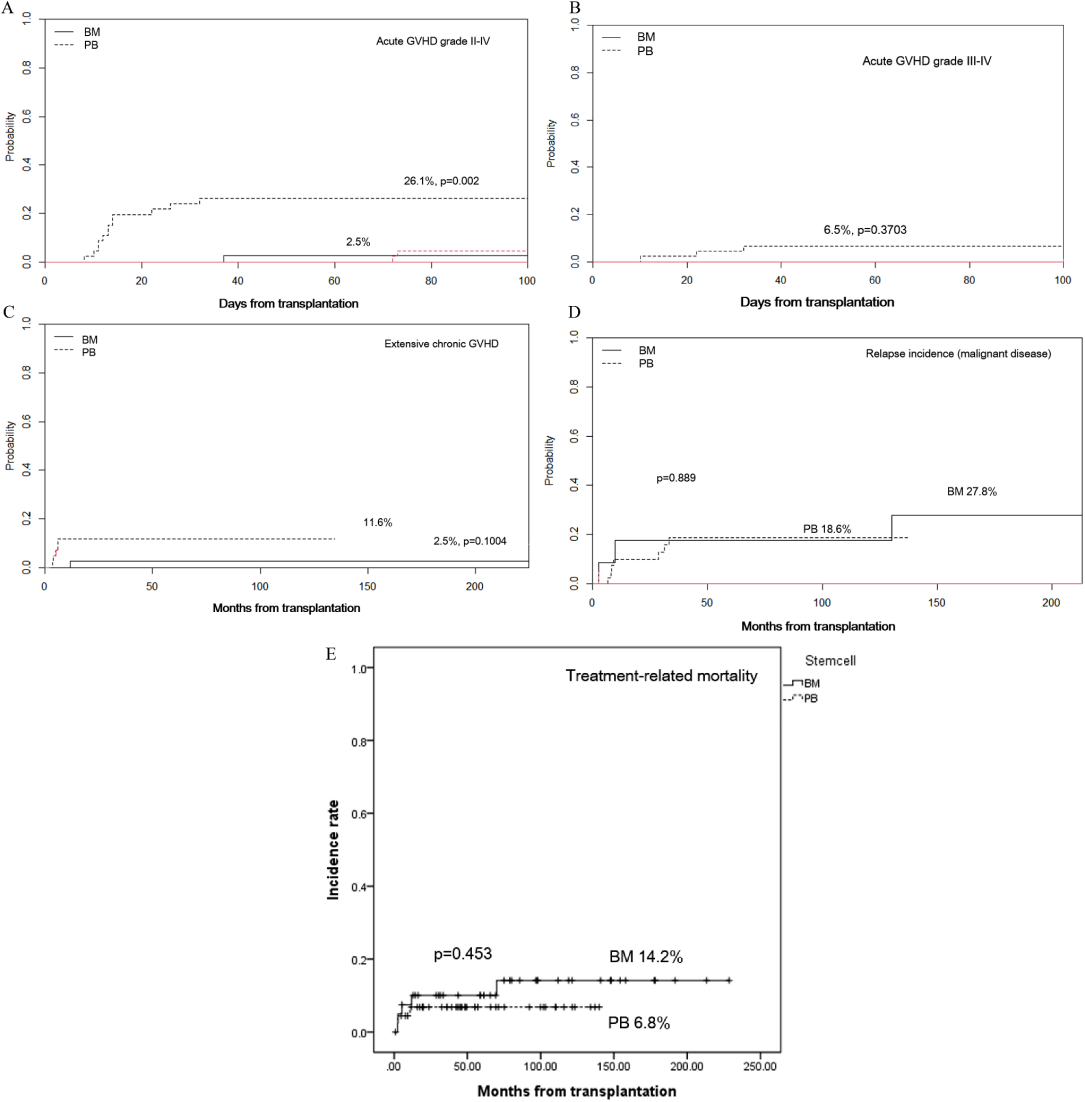
The CIs for (**A**) acute GVHD with grades II–IV were 2.5% for BM and 26.1% for PBSC (p = 0.002). For (**B**) acute GVHD with grades III–IV, the CIs were 0% for BM and 6.5% for PBSC (p = 0.3703). For (**C**) extensive chronic GVHD, the CIs were 2.5% for BM and 11.6% for PBSC (p = 0.1004). The CIs for (**D**) relapse incidence of malignant disease were 27.8% for BM and 18.6% for PBSC (p = 0.889), and for (**E**) TRM, the CIs were 14.2% for BM and 6.8% for PBSC (p = 0.453). CIs, cumulative incidences; GVHD; BM, bone marrow; PB, peripheral blood; TRM, treatment-related mortality

The CI of acute GVHD with grades II–IV was higher in PBSC (BM 2.5%, PBSC 26.1%, *p* = 0.002; Fig. 1A), but there was no significant difference in those of acute GVHD with grades III–IV (BM 0%, PBSC 6.5%, *p* = 0.3703; Fig. 1B) and extensive chronic GVHD (BM 2.5%, PBSC 11.6%, *p* = 0.1004; Fig. 1C). Particularly, patients with a high total nucleated cell (TNC) count (≥ 11.4 × 10^8^/kg) and high mononuclear cell (MNC) count (≥ 9.42 × 10^8^/kg) showed a high incidence of acute GVHD grades II–IV (TNC 26.3% vs. 5.26%, *p* = 0.012; MNC 27.0% vs. 5.13%, *p* = 0.009).

### Relapse, survival, and TRM

**Fig. 2:**
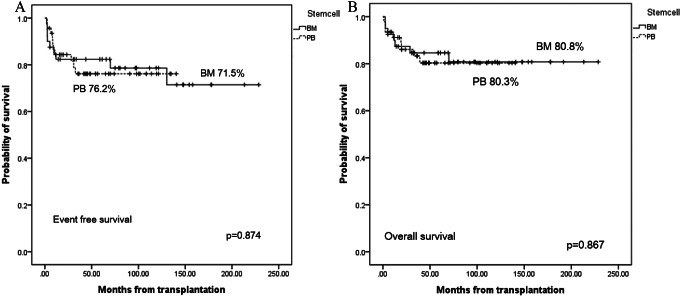
(**A**) The EFS rates were 71.5% for BM and 76.2% for PBSC (p = 0.874), and (**B**) the OS rates were 80.8% for BM and 80.3% for PBSC (p = 0.867). EFS, event-free survival; BM, bone marrow; PB, peripheral blood; OS, overall survival

There was no significant difference in the relapse incidence between BM and PBSC among patients with malignant diseases (BM 27.8%, PBSC 18.6%, *p* = 0.889; Fig. 1D. Additionally, there were no significant differences in the TRM (BM 14.2%, PBSC 6.8%, *p* = 0.453; Fig. 1E), 5-year EFS (BM 71.5%, PBSC 76.2%, *p* = 0.874), and OS (BM 80.8%, PBSC 80.3%, *p* = 0.867) rates between BM and PBSC in the univariate analysis (Fig. 2A and B). In the multivariate analysis, which included all factors with *p* < 0.50 in the univariate analysis, there was no significant prognostic factor for EFS or OS. Furthermore, there were no significant differences in the TRM, 5-year EFS, and OS rates between malignant and non-malignant diseases and between busulfan-based myeloablative regimens and reduced-intensity chemotherapy using cyclophosphamide. The causes of TRM included one case of lung GVHD, one case of fungal infection, and three cases of pneumonia among patients undergoing BMT. Among patients undergoing PBSCT, there were two cases of pneumonia and one case of sepsis.

### Donor complications

The median number of painkillers administered during hospitalization for SC collection was 1 (range, 0–4) in the BM and 0 (range, 0–5) in the PBSC (*p* = 0.017) groups. The median hospitalization period for SC collection was 4 days (range, 3–8) in the BM and 3 days (range, 2–10) in the PBSC (*p* = 0.276) groups. There were no long-term complications in either group and no significant difference in the rate of patients with side effects requiring an extension of the hospitalization period.

## Discussion

Recently, PBSCT has been increasingly used instead of BMT because of the easy collection of PBSCs, early engraftment, and cost-effectiveness [9–11]. However, PBSCT is yet to be established as a standard treatment because it has a higher incidence of GVHD than BMT, according to studies to date [12–15].

This study showed no significant differences in the EFS, OS, TRM, and relapse rates and GVHD, except for acute GVHD grades II–IV, between BMT and PBSCT from sibling donors using ATG (7.5 mg/kg). Most patients used cyclosporine and prednisolone for GVHD, whereas those who underwent PBSCT for malignant diseases used cyclosporine only for GVL. For this reason, the incidence of acute GVHD appeared to be higher in PBSCT, but there was no significant difference in acute GVHD grades III, IV.

Claudio Anasetti et al. [16] did not detect significant survival differences between PBSC and BM from unrelated donors; however, PBSC may reduce the risk of graft failure, whereas BM may reduce the risk of chronic GVHD. Ghavamzadeh et al. [17] reported that the survival advantage for BM compared with PB was not significant, and the rejection incidence was significantly lower in patients who used BM as their graft source. Acute and chronic GVHD were more frequent in PB, but the difference was not statistically significant.

Another study by Bacigalupo et al. [18] reported that BM should be the preferred SC source for matched sibling transplants in patients of all ages with acquired aplastic anemia. The survival advantage for recipients of BM rather than PB was statistically significant in patients aged 1–19 years (90% vs. 76%, *p* < 0.00001) as well as in patients aged over 20 years (74% vs. 64%, *p* = 0.001). Acute and chronic GVHD were more frequent in PB.

In our study, the incidence of acute GVHD grades II–IV was higher in PBSC, whereas the incidence of acute GVDH grades III–IV and chronic GVHD did not differ significantly, and the difference in the incidence of acute GVHD grades II–IV did not affect the mortality rate. The incidence of acute GVHD grades II–IV was higher in the group with a high TNC (≥ 11.4 × 10^8^/kg) and MNC (≥ 9.42 × 10^8^/kg) and was not related to the CD34 count. In a previous study, the CD34 cell dose in PBSC grafts appeared to affect the development of extensive chronic GVHD in matched sibling transplantation [19]. Injecting an appropriate amount of TNC, MNC, and CD34 is necessary to reduce the occurrence of GVHD.

No studies compared malignant and non-malignant diseases, so different conclusions have been drawn for each disease type. In this study, both malignant and non-malignant diseases were included and analyzed; however, the number of patients in each group varied significantly. Particularly, only three patients underwent PBSCT for non-malignant diseases, making analysis difficult; other studies have shown that BMT generally results in fewer cases of GVHD and is preferred for non-malignant diseases without the GVL effect. However, there is a burden on BMT donors during SC collection; particularly, parents are concerned about sibling donors. Additionally, the medical team may suffer from technical problems when collecting BM. In this study, there was no statistical difference regarding the number of hospitalization days or painkillers between BM and PB donors; however, BMT donors used painkillers more often, and the hospitalization period was longer [20].

PBSC is considered comparable to BM because new immunosuppressants are emerging and can prevent and treat severe GVHD. There were no significant differences in EFS, OS, TRM, and GVHD, except for acute GVHD grades II–IV between BMT and PBSCT from sibling donors, using ATG (7.5 mg/kg). Therefore, the collection of PB, which is less invasive for donors and less labor-intensive for doctors, could also be considered an acceptable SC source for sibling donor HSCT in children.
